# Massive atelectasis by mucoid impaction in an asthma patient during treatment with anti‐interleukin‐5 receptor antibody

**DOI:** 10.1002/rcr2.599

**Published:** 2020-06-18

**Authors:** Takayuki Takimoto, Tomoko Kagawa, Kazunobu Tachibana, Toru Arai, Yoshikazu Inoue

**Affiliations:** ^1^ Department of Internal Medicine National Hospital Organization Kinki‐Chuo Chest Medical Center Osaka Japan; ^2^ Clinical Research Center National Hospital Organization Kinki‐Chuo Chest Medical Center Osaka Japan

**Keywords:** Asthma, atelectasis, benralizumab, interleukin‐5, mucus hypersecretion

## Abstract

Benralizumab is an interleukin‐5 (IL‐5) receptor α‐directed cytolytic monoclonal antibody that reduces rapid and nearly complete depletion of eosinophils by enhancing antibody‐dependent cell‐mediated cytotoxicity. The depletion of eosinophilic inflammation is expected to reduce mucus hypersecretion and mucoid impaction. A 75‐year‐old non‐smoking female had been treated for uncontrolled bronchial asthma with multiple drugs. Treatment with benralizumab was initiated after the asthma attack; however, four months later, she developed massive atelectasis in the left lung leading to the tracheal deviation, to the extent that nasal high‐flow therapy was required. The laboratory data showed elevated neutrophil count, whereas blood eosinophils were almost completely depleted. The thick mucus was removed by bronchofiberscopy and the atelectasis was completely resolved. No exacerbation has been observed for nine months after discontinuation of benralizumab and initiation of erythromycin. This is the first documented case that developed atelectasis by mucoid impaction during treatment with anti‐IL‐5 receptor antibody.

## Graphical abstract

We herein report a case of a patient who developed massive atelectasis by mucoid impaction during treatment with anti‐interleukin (IL)‐5 receptor antibody.

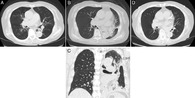

## Introduction

Asthma remains a significant cause of mucoid impaction of the airways and mortality. Mucoid impaction results from increased mucus production, which is often caused by eosinophilic inflammation in asthma, as is typically seen in allergic bronchopulmonary aspergillosis (ABPA). Benralizumab is an interleukin‐5 (IL‐5) receptor α‐directed cytolytic monoclonal antibody that reduces rapid and nearly complete depletion of eosinophils by enhancing antibody‐dependent cell‐mediated cytotoxicity, which is an apoptotic process of eosinophil elimination [1]. The depletion of eosinophilic inflammation is expected to reduce mucus hypersecretion and mucoid impaction; however, we herein report a case of a patient who developed massive atelectasis by mucoid impaction during treatment with anti‐IL‐5 receptor antibody.

## Case Report

**Figure 1:**
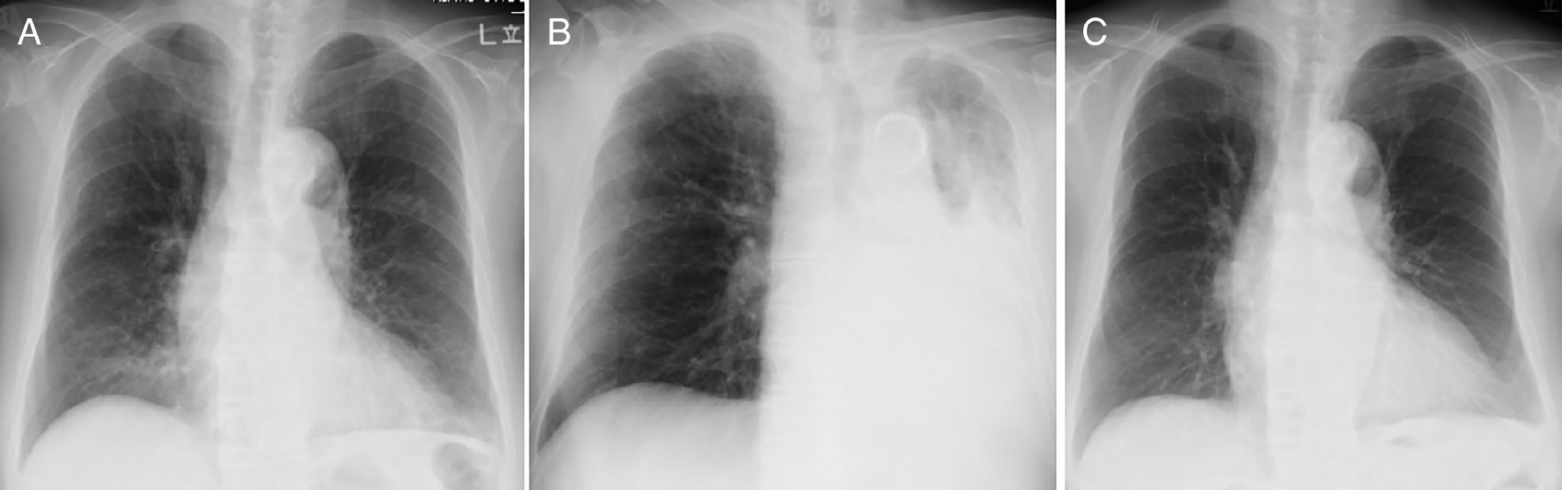
(A) Chest X‐ray at initial visit. (B) Chest X‐ray on exacerbation of the atelectasis, leading to the tracheal deviation. (C) Chest X‐ray showing a complete resolution of the atelectasis.

**Figure 2:**
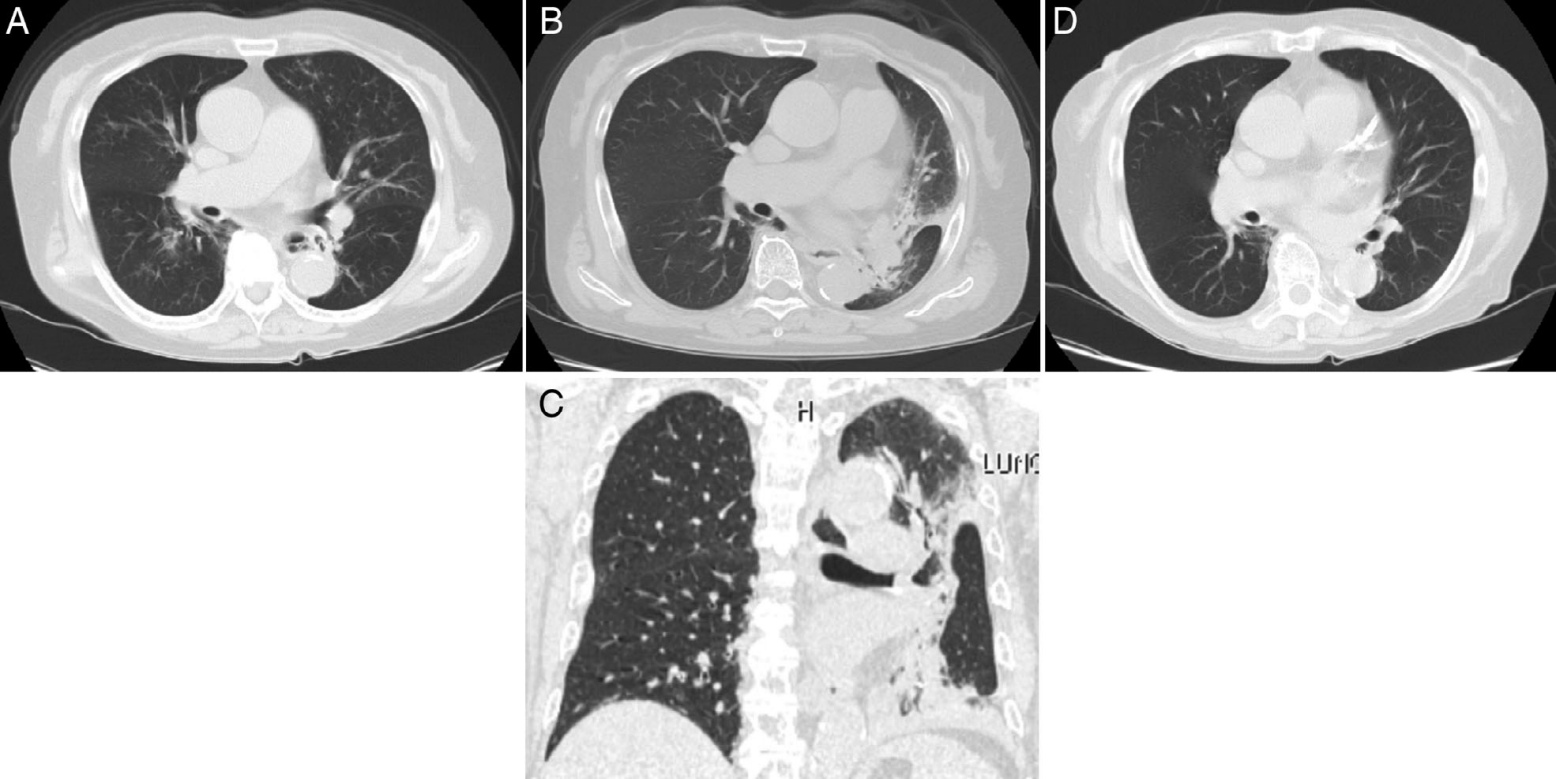
(A) Thoracic computed tomography (CT) at initial visit. Transverse (B) and coronal (C) view of thoracic CT on readmission, showing atelectasis by mucoid impaction in the left lung. (D) Thoracic CT on day 17 of readmission, showing a complete resolution of the atelectasis.

A 75‐year‐old non‐smoking female patient was referred to our hospital for an asthma attack. She had been previously treated for uncontrolled bronchial asthma with multiple drugs, including inhaled corticosteroids, long‐acting β‐agonists, and leukotriene receptor antagonists, for 28 years. She had allergic rhinitis and sinusitis, but not nasal polyps or atopic dermatitis. At initial visit, wheezes were heard on auscultation. The laboratory data showed an elevated C‐reactive protein level (7.24 mg/dL) and neutrophil count (9510/μL). The blood eosinophil count, serum immunoglobulin (Ig) E, and fractional exhaled nitric oxide (FeNO) were 210–692/μL, 159 IU/mL, and 28 ppb, respectively. Specific IgE and IgG to Aspergillus and anti‐neutrophil cytoplasmic antibodies were negative. The forced expiratory volume in 1 sec was 1.18 L (FEV_1_%: 65.6%). Chest X‐ray (Fig. 1A) and thoracic computed tomography (CT) (Fig. 2A) demonstrated bronchial wall thickening and centrilobular nodules diffusely in both lungs, without central bronchiectasis. Following treatment with antibiotics and systemic corticosteroids, treatment with benralizumab was initiated.

Four months later, she was readmitted to our hospital for severe respiratory failure. Physical examination revealed decreased respiratory sounds in the left lung. Thoracic CT (Fig. 2B, C) demonstrated atelectasis by mucoid impaction in the left lung. The laboratory data showed elevated C‐reactive protein level (9.25 μg/dL) and neutrophil count (8310/μL). Blood eosinophils were almost completely depleted, and the serum IgE level was not elevated. Pathogens, including bacteria and fungus, and Charcot–Leyden crystals were not detected in the sputum (0.75% of eosinophil counts). Systemic corticosteroids, antibiotics, and expectorants were administered; however, her respiratory condition exacerbated on the next day, due to the massive atelectasis leading to the tracheal deviation, to the extent that nasal high‐flow therapy was required (Fig. 1B). The thick mucus was removed from the left main bronchus and the lower lobe bronchi by bronchofiberscopy and a complete resolution of the atelectasis was confirmed by chest X‐ray (Fig. 1C) and thoracic CT (Fig. 2D) on day 17 of readmission. No exacerbation has been observed for nine months after discontinuation of benralizumab and initiation of erythromycin.

## Discussion

This is the first documented case of a patient who developed atelectasis by mucoid impaction during treatment with an anti‐IL‐5 receptor antibody. Benralizumab treatment nearly completely depleted the eosinophils in blood, which is consistent with the previous report [2]. In that report, benralizumab produced decrease from baseline of 95.8% in airway mucosal eosinophils, 89.9% in sputum, and 100% in blood, 12 weeks after treatment. These data raise the intriguing question: What is the cause of mucus development during anti‐IL‐5 therapy?

Eosinophilic inflammation has a pivotal role in mucus plug formation. Recent evidence highlighted the eosinophil‐derived cytolytic extracellular trap cell death (ETosis) in the formation of eosinophilic mucoid impaction, especially in ABPA. The activated eosinophils can release extracellular chromatin to form DNA traps through ETosis. In comparison with neutrophil‐derived ETosis, eosinophil‐derived ETosis is mediated through the induction of thicker fibres with globular structures. Nevertheless, the role of eosinophils in mucus hypersecretion and airway hyperresponsiveness is still controversial on the basis of studies involving eosinophil‐deficient mice in an asthma model [3].

Asthma is increasingly recognized as a heterogeneous disease involving multiple phenotypes and endotypes, such as eosinophilic or non‐eosinophilic [4]. Neutrophilic inflammation is associated with more severe subtypes of asthma. In clinical trials, macrolide antibiotics, which is known to inhibit neutrophil function, are suggested to be beneficial in persistent uncontrolled asthma, especially in non‐eosinophilic asthma (AZISAST; AMAZES). Therefore, controlling neutrophilic inflammation may be important for suppressing mucus hypersecretion and preventing exacerbation in some patient populations. Regarding the mechanisms underlying mucus hypersecretion, activated neutrophils recruited to the airways along with their secreted products, including neutrophil elastase and reactive oxygen species, play key roles in mucus hypersecretion in severe asthma. Recently, extracellular DNA, neutrophil‐derived ETosis, and inflammasome activation are implicated in severe asthma [5].

There are several possible mechanisms underlying mucus development during anti‐IL‐5 therapy. First, residual eosinophils in the airways even after anti‐IL‐5 therapy can have the effect on mucus development. In an animal study, it is speculated that eosinophil deficiency was not protective against mucus hypersecretion, possibly due to residual lung eosinophils [3]. Second, eosinophil‐independent pathways can be involved, as previously described. With regard to the present case, the laboratory data indicated an exacerbation of neutrophilic inflammation under the condition that eosinophils were nearly completely depleted by benralizumab. In addition, thereafter, macrolide antibiotics, which is known to inhibit neutrophil function, might have prevented mucoid impaction or exacerbation for at least nine months. The mucoid impaction could be caused either by non‐eosinophilic, and possibly, neutrophilic inflammation, or a bacterial infection. Controlling eosinophilic inflammation might not be enough to prevent mucus hypersecretion and exacerbation in some patient populations. Third, the consecutive side effect of eosinophil depletion can be considered. However, deficiency of eosinophils is not associated with any characteristic abnormality both in human diseases and genetically manipulated animals. Regrettably, the precise mechanism for mucus development during anti‐IL‐5 therapy remains unclear, because sputum samples of pre‐ and post‐treatment with benralizumab were not available.

In conclusion, we described a case of a patient who developed massive atelectasis by mucoid impaction during benralizumab treatment. Anti‐IL‐5 therapy may not be enough to prevent mucus hypersecretion. Further research into non‐eosinophilic or neutrophilic inflammation is warranted to better understand the potential benefit or risk of anti‐IL‐5 therapy, based on asthma phenotypes.

### Disclosure Statement

Appropriate written informed consent was obtained for publication of this case report and accompanying images.
